# Personal

**Published:** 1917-04

**Authors:** 


### PERSONAL

Announcement of removal, travel, and other matters of interest are requested. Please report errors in the listing of any physician in the State and other directories, that we may co-operate with the State Society in securing a correct list.

---

Dr. J. L. Mangano has been appointed Assistant Medical School Inspector for Buffalo.

---

Dr. Frank E. Brundage of Buffalo, announces the removal of his residence and office to 572 W. Ferry St.

---

Dr. Monroe Manges of Buffalo has returned from a trip to Pass Christian, Miss.

---

Dr. Timothy E. Donovan of Buffalo announces the removal of his office to Delaware Court, 250 Delaware Ave.

---

Dr. Francis M. O'Gorman has recently been appointed attending surgeon at "The Buffalo Hospital of the Sisters of Charity."

